# Elements of Materia Medica

**Published:** 1834-04-01

**Authors:** 


### Elements of Materia Medica and Therapeutics, including the recent Discoveries and Analyses of Medicines. By *A. T. Thomson*, M.D., &c. Second volume, 1834.

We gave a short notice of the first volume of this valuable work, several months ago, and we now proceed to the second and concluding volume. Few medical practitioners have devoted so much time and labour to materia medica as Dr. Thomson, while the circumstance of his being a physician of ample experience and accurate observation, qualified him well for the second, and not less important subject, the application of medicines to the treatment of diseases. In a dispensatory the subject of therapeutics can scarcely be touched upon; and even in such a work as that before us, the author has but little scope for the introduction of personal observation and remark on the difficult art of applying remedies to the living machine. It may be said that therapeutics must be *practical* and nothing else. This is an error. There are more theories at the bed-side than in the closet. Very few even of our oldest practitioners feel the pulse or examine the tongue of a patient, without a theory—too often a prejudice, starting into his mind, and influencing the prescription which he writes. Dr. Thomson is far from being free from these *tendencies*, almost inseparable from the human mind—and to which, indeed, strong minds are much more prone than weak ones.

The work being almost entirely elementary, and adapted to the medical student, we cannot attempt anything like an analysis—and the author has laid in too large a stock of reputation to require criticism on a work where he is so much at home on all chemical and pharmaceutical points. We shall only therefore give two or three short extracts to shew the manner in which the work is executed.

1. *Ratanhy Root.* In combination with purified animal charcoal, in the proportion of one part to three of charcoal, it forms the best tooth-powder that can be produced.

2. *Rumex Aquatiens—Great Water-Dock.* Dr. T. has had no experience of this except in two diseases—both of which are very difficult to cure—viz. herpes labialis, when it changes into a species of impetigo—and ichthyosis, or fish-skin eruption.

3. *Ruspini's Styptic* owes its powers, according to Dr. T. to gallic acid, combined with a small proportion of sulphate of zinc and opium, dissolved in a mixture of alcohol and rose-water. We confess that we doubt this, as we have been able to perceive neither taste nor smell in some samples of this styptic, which proved very efficacious, nevertheless, in two alarming cases of internal hemorrhage.

4. From our author's dissertation on the application of astringents in the treatment of diseases, we shall make a short quotation.

"In hæmoptysis, if the excitement be considerable, after bleeding at the arm, the use of cold water and of ice, with binacetate of lead in combination with diluted acetic acid, are indicated. When the effusion of blood from the lungs is considerable, no circumstances should interfere to prevent us from endeavouring immediately to check it: but when it is not of this alarming character, and there is no obvious predisposition to tubercular consumption, especially if it be the consequence of a suppression of the menstrual discharge, it should only be moderated, not checked suddenly, which might induce a congestion in some organ less capable of supporting it with impunity. The kind of Astringent most resorted to in this form of hæmorrhage is, as I have already said, the binacetate of lead; and I have no hesitation in recommending it either alone or in conjunction with opium, in much larger doses than it has hitherto been given. Two drachms of it have been swallowed accidentally, instead of lump sugar, without any serious evil resulting—a constriction of the œsophagus and costiveness being the sole effects experienced. It is necessary to remark, that, when it is given in the fluid state with laudanum, vinegar should be added to redissolve the morphia, which is thrown down with the meconate of lead. Indeed, in every case, the remedy is more safe when given in conjunction with diluted acetic acid, which prevents its conversion into the carbonate of lead." 85.

5. Under the head of errhines, we have the following observations on snuff-taking.

"Snuffing is the most frequent and the least injurious mode of using Tobacco; although, in those unaccustomed to it, it causes nausea and vertigo. In great snuffers the stomach frequently suffers; dyspeptic symptoms supervene, accompanied with pains and a sensation of twisting in the bowels—effects which may result from the snuff passing into the pharynx and being swallowed; although it is also possible that they may proceed from sympathy. Instances daily occur, in which quantities of snuff are coughed up by great snuff-takers; and Dr. Alston says that some persons have thrown up balls of snuff. It is generally injurious in weak and what are termed nervous subjects; and some practitioners, among whom is the celebrated Lorry, have ascribed the more frequent occurrence of nervous diseases to the daily excessive taking of snuff. Upon the whole, however, it is probable that the statements respecting the baneful effects of snuff, are, at least, greatly exaggerated. In the manufactories of snuff in France, in which upwards of 4000 persons are employed, it has been ascertained that the workmen become habituated to the atmosphere of the manufactory; that they are neither subject to special diseases, nor to disease generally; and that they live, on an average, as long as other tradesmen." 122.

An amusing anecdote is related of Fagou, a celebrated physician of Louis XIV., who, in the midst of an impassioned oration on the pernicious effects of snuff-taking, made a pause, and, in an unguarded moment, took out his box and treated himself to a hearty pinch—causing no small merriment among his auditors!

6. Passing over many sections, containing highly useful matter for students, but not calculated for notice in a Journal of this kind, we extract the following passage respecting liquor potassæ as a diuretic.

"As it acts (potassa) as a powerful escharotic when applied to the body, it cannot be administered internally in a solid form; it must be largely diluted with water, or some bland, aqueous fluid. In the form of Liquor Potassæ of the London Pharmacopœia, it may be given in very large doses, if the dose be gradually augmented and sufficiently diluted. This solution, when properly prepared, is limpid, colourless, and void of any odour: it is too acrid and caustic to be tasted alone; and, when rubbed between the fingers, it feels soapy, owing to a solution of the cuticle. Its specific gravity should be 1.056; and one pint of it should weigh sixteen ounces. It ought not to effervesce with acids, nor to render lime-water turbid; but it is seldom prepared for medicinal purposes so free from carbonic acid, nor is this perfection requisite. When it is desirable to free it entirely from carbonic acid, the alkaline solution and the lime should be boiled together.

In its ordinary state, Liquor Potassæ, when given in doses of from ten minims to f℥ss. in a large cupful of water, or of milk, or almond emulsion, or even beer if it be not sour, passes unaltered to the kidney and acts as a Diuretic. When taken into the stomach, however, its primary effect is upon that viscus. If any acid be present, it neutralizes it; and when the dose is sufficiently large to allow the alkali to predominate, or if no acid be present, it then acts as a sedative upon the stomach, allaying its morbid irritability, and enabling it to secrete the gastric juice more slowly, and consequently in a more healthy state. Its secondary action is upon the kidney, to which it is carried undecomposed; and its passage through which can be detected by testing the urine with infusion of rhubarb or of turmeric. As a Diuretic, Potassa is not extensively employed, owing to a fallacious opinion that it is injurious, if given in doses sufficient to produce its full effect. I have given it to the extent of upwards of 100 drops, three times a day, in psoriasis, without any bad effect; but, on the contrary, with the greatest advantage. It must not, however, be given in such doses at first; but, beginning with ten or fifteen drops, the dose should be gradually increased until the full dose be administered. As soon as it displays its diuretic effects, the influence of the remedy over the disease becomes obvious: but its use must be continued, and the dose as gradually diminished as it was increased, for some time after the disease has disappeared." 397.

We strongly recommend Dr. Thomson's work both to students and practitioners, as an excellent reference on the important subjects of materia medica and therapeutics.

